# Post-Streptococcal Antibodies Are Associated with Metabolic Syndrome in a Population-Based Cohort

**DOI:** 10.1371/journal.pone.0025017

**Published:** 2011-09-20

**Authors:** Adi Aran, Ling Lin, Laurel Ann Finn, Karin Weiner, Paul Peppard, Terry Young, Emmanuel Mignot

**Affiliations:** 1 Psychiatry and Behavioral Sciences, Stanford University, Stanford, California, United States of America; 2 Department of Population Health Sciences, University of Wisconsin, Madison, Wisconsin, United State of America; 3 Shaare Zedek Medical Center, Jerusalem, Israel; Indian Institute of Science, India

## Abstract

**Background:**

Streptococcal infections are known to trigger autoimmune disorders, affecting millions worldwide. Recently, we found an association between post-streptococcal autoantibodies against Protein Disulphide Isomerase (PDI), an enzyme involved in insulin degradation and insulin resistance. This led us to evaluate associations between post-streptococcal antibodies and metabolic syndrome, as defined by the updated National Cholesterol Education Program definition, 2005.

**Methods and Findings:**

Metabolic data (HDL, triglycerides, fasting glucose, blood pressure, waist circumference, BMI, smoking), post-streptococcal antibodies (anti-Streptolysin O (ASO) and anti-PDI), and C-reactive protein (CRP, as a general inflammatory marker), were assessed in 1156 participants of the Wisconsin Sleep Cohort Study. Anti-PDI antibodies were found in 308 participants (26.6%), ASO≥100 in 258 (22.3%), and 482 (41.7%) met diagnostic criteria for metabolic syndrome. Anti-PDI antibodies but not ASO were significantly associated with metabolic syndrome [n = 1156, OR 1.463 (95% CI 1.114, 1.920), p = 0.0062; adjusted for age, gender, education, smoking]. Importantly, the anti-PDI - metabolic syndrome association remained significant after adjusting for CRP and fasting insulin.

**Conclusions:**

Post-streptococcal anti-PDI antibodies are associated with metabolic syndrome regardless of fasting insulin and CRP levels. Whereas these data are in line with a growing body of evidence linking infections, immunity and metabolism, additional studies are necessary to establish the post-streptococcal – metabolic syndrome association.

## Introduction

Metabolic syndrome is characterized by a cluster of metabolic risk factors for cardiovascular diseases and type 2 diabetes that include abdominal obesity, dyslipidemia, hypertension, insulin resistance, prothrombotic and proinflammatory state. Other conditions associated with the syndrome include physical inactivity, aging, hormonal imbalance and genetic predisposition. Approximately 25% of the adult US population meet criteria for the metabolic syndrome [1], [2].

In a recent study, we reported that a subset of patients with post-streptococcal immunity, as defined by the presence of anti-Streptolysin O (ASO) or anti-DNase B (ADB) antibodies also carry auto-antibodies against Protein Disulfide Isomerase (PDI), a pleiotropic enzyme. We found that these auto-antibodies neutralize PDI, decrease insulin degradation and correlate with higher insulin levels and insulin resistance [3]. PDI catalyzes disulfide bonds formation, breakage and rearrangement [4]. Extracellular PDI is involved in several metabolic pathways including insulin degradation [5], [6], platelet aggregation and secretion [7], fibrin formation [8], [9], [10] and intracellular nitric oxide delivery [11]. In addition to its role in disulfide bond formation, PDI is involved in regulating NAD(P)H oxidase [12] and is also the smaller subunit of microsomal triglyceride transfer protein which catalyzes the assembly of apoB containing lipoproteins in the liver and intestinal cells [13]. Cell surface PDI can also bind estradiol and tri-iodotyronine and may impact on the hormone-receptor interaction [14].

Interestingly, two types of anti-PDI autoantibodies were found in our prior study, one with higher affinity for the human PDI versus the bovine PDI protein, and a second antibody with the opposite profile. Although the presence of these antibodies correlated only partially with each other, they both were highly correlated with ASO, suggesting that they are part of a spectrum of common post-streptococcal immune response [3]. Furthermore, we found that the binding sites of the bovine and human PDI have a strong structural similarity with a specific sequence in the streptococcal toxin Streptolysin O indicative of molecular mimicry, initiated by the ASO response.

In the current study, we further assessed associations between immunity and metabolism and explored whether post-streptococcal immune status is associated with metabolic alterations beyond insulin resistance.

## Methods

### Ethics Statement

The institutional review board of the University of Wisconsin Medical School approved all protocols for the study and a written informed consent was obtained from all participants.

### Subjects

The Wisconsin Sleep Cohort Study is an ongoing longitudinal study in which metabolic, sleep data and blood samples are obtained every four years for each participant [15].

Briefly: to construct a defined sampling frame, all employees aged 30–60 y of four state agencies in south central Wisconsin were mailed in 1989 a survey on sleep habits, health, and demographics. Mailed surveys were repeated at 5-y intervals and based on that survey new participants were added between 1989–2000. A 71% response rate (n = 4896) was obtained from this mailed survey. A stratified random sample of respondents (n = 3028) was then recruited. Stratification was based on risk for sleep-disordered breathing (SDB), with an oversampling of habitual snorers to ensure an adequate distribution of SDB. Exclusion criteria included pregnancy, unstable cardiopulmonary disease, airway cancers, and recent upper respiratory tract surgery. The baseline response rate was 51% (n = 1545), with most refusals due to the inconvenience of sleeping away from home required for the polysomnogram. Collection of morning, fasted blood was added to the protocol in 1993. Follow-up studies have been conducted at 4-y intervals. Data to define metabolic syndrome was collected at all overnight studies (baseline and at the 4 year follow-ups) including: measurements of waist girth and blood pressure, an interview where participants report prescription drug use and doctor diagnosed diabetes and blood samples were obtained for triglycerides, HDL and glucose. Insulin, CRP and post-streptococcal antibodies were assessed at a later date on banked sera. Extensive survey and other data available from the sampling frame have been used to evaluate the potential for response and drop out biases. The most recent 4 year follow-up for this paper was in 2009 and the average 4 year follow-up participation rates are 80%; among those lost to follow up were 104 deceased individuals, 17 due to undeliverable addresses, 6 who asked not to be called again and 5 for medical reasons. All available visits (serum samples) were used for the current study and included a total of 2,471 visits of 1156 participants. [Mean age at blood draw 55±8.6 (SD); range 34.9–78.2 years; 45.7% females, 97.1% Caucasians, 1.1% Asians, 0.9% African -Americans].

### Metabolic syndrome

The criteria used to diagnose metabolic syndrome were those proposed by the National Cholesterol Education Program (NCEP) Adult Treatment Panel III (ATP III), and revised by the American Heart Association (AHA) and the National Heart, Lung, and Blood Institute (NHLBI) [16]. At least three of the following criteria were mandatory for diagnosis: (a) Elevated waist circumference: Men: ≥102 cm ; Women: ≥88 cm; (b) Elevated triglycerides: ≥150 mg/dL or on drug treatment for elevated triglycerides; (c) Reduced HDL cholesterol: Men: <40 mg/dL; Women: <50 mg/dL or on drug treatment for reduced HDL-C ; (d) Elevated blood pressure: ≥130/85 mm Hg or on antihypertensive drug treatment in a patient with a history of hypertension; (e) Elevated fasting glucose: ≥100 mg/dL or on drug treatment for elevated glucose.

### Antibodies and reagents

Bovine PDI and rabbit polyclonal anti-bovine PDI antibody (cross-reacting with rat and human PDI) were obtained from Invitrogen. Recombinant human PDI protein was obtained from Assay Designs.

#### Assays

*ASO* and *CRP* were assessed using commercially available kits (SeraTest ASO, Remel KS, USA; CRP ELISA, Alpha Diagnostic International, TX, USA) according to the manufacturer's instructions.

#### Anti-PDI score

The presence of anti-human and anti-bovine PDI autoantibodies were determined using in house ELISA, and subjects positive for either human or bovine PDI were considered PDI positive. Microtiter plates (96 wells) were coated with bovine (100 µl of 1.5 µg/mL) or recombinant human (100 µl of 5 µg/mL) PDI (incubated at 4°C, 24 hours). Plates were rinsed and flicked 3× (0.05% Triton in PBS), blocked (5% nonfat dry milk in PBS), and exposed to human sera diluted in 5% milk (90 min, room temperature). Anti-bovine PDI antibodies were detected using a 1∶250 dilution; anti-human PDI antibodies using a 1∶50 dilution. These dilutions are compatible with high and low affinity antibodies respectively. Donkey anti-human IgG-HRP (Jackson ImmunoResearch Laboratories, 1∶10,000) was used as the secondary antibody. To detect antibody–antigen interactions tetramethylbenzidine (TMB, Vector Laboratories) was added (reaction stopped at 5 min with 50 µl H3PO4 (1 M)), and the resulting signal read at 450 nm (Molecular Devices EMAX micro plate reader). The cutoff for positive was OD≥0.5 for anti-bovine PDI and OD≥0.75 for anti-human PDI (due to higher background). All samples were assayed in duplicate.

### Statistical analysis

All analyses were performed with SAS software (version 8.02; SAS Institute Inc, Cary, NC). For comparison of demographic characteristics between the PDI positive and negative individuals, we used t-tests and Chi-square tests. For modeling continuous outcomes, such as waist girth mean, we use generalized linear models and PDI status as the predictor variable, adjusted for age, sex, smoking status, and education. There was one continuous variable that was not normally distributed. For this variable, CRP, we used a log transformation. For modeling binary variables, such as metabolic Syndrome, we used logistic regression with PDI status as the predictor, adjusted for age, sex, smoking status, and education. Resulting estimated effects (odds ratios/least square means) were calculated from the beta coefficients of these models. P-values of 0.05 were considered statistically significant.

## Results

### Anti-PDI is associated with metabolic syndrome

Table 1 includes the characteristics of the cohort sample at baseline (n = 1156 subjects), stratified by anti-PDI status. Antibodies against bovine or human PDI were found in 26.6% of the subjects (n = 308) with some predominance for younger individuals, males and higher socio-economic status. Participants who were positive for anti-PDI antibodies were more likely to meet the diagnostic criteria for metabolic syndrome [OR 1.463 (95% CI 1.114, 1.920), p = 0.0062; adjusted for age, gender, education, smoking].

**Table 1:** Baseline demographic and metabolic characteristics, stratified by anti-PDI status (n = 1156 subjects).

	*Negative for anti-PDI* * *(n = 848)*	*Positive for anti-PDI* † *(n = 308)*	*p-value*
**Age**	52.7 (7.9)	52.0 (7.0)	0.1354
**Male Sex**	454 (54%)	171 (56%)	0.5500
**BMI**	31.0 (6.8)	31.4 (7.0)	0.3858
**Smoking**			
**Never**	400 (47%)	164 (53%)	0.1625
**Past**	325 (38%)	101 (33%)	
**Current**	123 (15%)	43 (14%)	
**At least some college education**	632 (75%)	236 (77%)	0.4665
**Abdominal obesity** ‡	REF	1.26 (0.96, 1.66)	0.0925
**Raised Triglycerides** ‡	REF	1.21 (0.93, 1.58)	0.1636
**Reduced HDL** ‡	REF	1.37 (1.05, 1.79)	0.0199
**Hypertension** ‡	REF	1.29 (0.98, 1.69)	0.0685
**Raised fasting glucose** ‡	REF	1.33 (1.01, 1.76)	0.0457
**Metabolic Syndrome** ‡	REF	1.46 (1.11, 1.92)	0.0062
**Log (CRP (mg/dL))** ‡ ^,^ §	1.08 (0.04)	1.12 (0.06)	0.5957
**CRP>3 mg/dL** ‡ ^,^ §	REF	1.01 (0.75, 1.36)	0.9440
**CRP>10 mg/dL** ‡ ^,^ §	REF	1.03 (0.61, 1.74)	0.9246
**Insulin (iu/ml)** ‡ ^,^ §	12.0 (0.37)	12.3 (0.60)	0.6078

Results are presented as: mean (SD), number of subjects (%) or as Odds Ratio (95% Confidence Interval).

*Negative for both human PDI and Bovine PDI.

† Positive for bovine PDI and/or human PDI.

‡ Adjusted for age, sex, education, and smoking.

§ Reduced sample size (n = 985).

Significant associations between anti-PDI antibodies status and single components of metabolic syndrome were found for reduced HDL-C and raised fasting glucose.

CRP results were available for 985 of the 1156 participants. Both mean CRP values and positive status were not higher in anti-PDI positive participants (Table 1), suggesting a specific immune response against PDI rather than a generalized immune activation. The association between anti-PDI antibodies and metabolic syndrome in these 985 subjects was significant after adjusting for age, gender, education and smoking [OR 1.5 (95% CI 1.1, 2.0), p = 0.0116]. Adjusting the results also for log (CRP) didn't have a noticeable effect [OR 1.5 (95% CI 1.1, 2.0), p = 0.0176], indicating that CRP is not a significant mediator in the anti-PDI – metabolic syndrome association.

Of 1156 participants, 1118 subjects had a second assessment in a 4 year interval. More than half of the subjects with positive anti-PDI status in the first visit were still positive in the second visit 4 years later (Table 2), suggesting a relatively long lasting process with a potential for prolonged metabolic alterations.

**Table 2:** Stability of anti-streptococcal antibodies status across time.

*First visit results*	*Second visit results*			
	*ASO<100*	*ASO≥100*	*Anti-PDI negative* *	*Anti-PDI positive* †
***ASO<100 (n = 879)***	831 (95%)	48 (5%)		
***ASO≥100 (n = 239)***	154 (64%)	85 (36%)		
***Anti-PDI negative*** * ***(n = 824)***			739 (90%)	85 (10%)
***Anti-PDI positive*** † ***(n = 294)***			134 (46%)	160 (54%)

1118 individuals had 2 subsequent visits in a 4-year interval. Results in the second visit are stratified by the first visit results.

*Negative for both human PDI and Bovine PDI;

† Positive for bovine PDI and/or human PDI.

### The anti-PDI – metabolic syndrome association is not dependant on fasting insulin values

In a former study we demonstrated an association between anti-human PDI antibodies, fasting insulin and insulin resistance [3]. In the current study we found an association between anti-PDI (human and/or bovine) and metabolic syndrome. Adjusting the results for fasting insulin had no effect on the association [n = 985, OR (95% CI) = 1.5 (1.1, 2.1), p = 0.0145, compared to OR (95% CI) = 1.5 (1.1, 2.0), p = 0.0116 before adjustment], suggesting that anti-PDI antibodies are associated with metabolic alterations beyond insulin resistance.

### Anti-Streptolysin O (ASO) is not significantly associated with metabolic syndrome

At the first visit of the 1156 subjects, ASO≥100 was found in 258 cases (22.3%) with mild predominance in younger age and women. A value over 100 IU was considered positive for this age group [17]. Participants who were ASO positive had some tendency toward lower HDL and larger waist circumference and were more likely to meet the diagnostic criteria for metabolic syndrome but the results were not significant [OR 1.116 (95% CI 0.835, 1.491), p = 0.4597, adjusted for age, gender, education and smoking]. CRP values were not significantly more elevated in ASO positive subjects.

As previously reported [3], we found that the presence of anti-PDI antibodies (human or bovine PDI) correlated with ASO (Kappa = 0.2443, p<0.0001, n = 2471). At the first visit of the 1156 subjects, 119 (10.3%) were positive for both post-streptococcal antibodies: anti-PDI and ASO, 328 (28.4%) were positive for one of them, and 709 participants (61.3%) were negative for both antibodies. Combinations of anti-PDI and ASO (Participants who were positive for both or those who were positive for at least one, compared to participants who were negative for both) did not demonstrate a greater association with metabolic syndrome compared to only anti-PDI.

## Discussion

In this study we found a positive association between the presence of post-streptococcal antibodies (anti-bovine PDI and/or anti-human PDI) and metabolic syndrome. The association was independent of socioeconomic correlates, CRP as a marker of general immune activation and fasting insulin levels. This study bolsters our former finding of positive association between anti-human PDI and insulin resistance, an association we postulated to be due to the inhibition of PDI mediated insulin degradation [3].

This is also not the first time that metabolic syndrome is suggested to have a post infectious component. Several pathogens have been reported to cause obesity in animal models [18], [19], and Vijay-Kumar et al suggested interactions between the immune system and the gut microbiota as both necessary and sufficient for the development of metabolic syndrome in mice [20]. These findings are also in line with the well known importance of inflammatory mechanisms in metabolic syndrome [21], [22], [23], [24]. Indeed, though this study indicates that metabolic syndrome could have a post-streptococcal component, other infections might be involved as well and should be assessed in future studies.

Our results suggest that anti-PDI antibodies are associated, to a certain degree, with each of the 5 components in the definition of metabolic syndrome, but significant associations were found only with reduced HDL-C and raised fasting glucose. The mechanism by which post-streptococcal immunity alters the risk of metabolic syndrome and its individual components is unknown and could be multi-factorial. Nonetheless, our results suggest that autoimmunity directed toward the PDI enzyme may be one of the mechanisms involved, and considering the pleiotropic role of PDI, it is an intriguingly strong candidate. PDI is a multifunctional enzyme that regulates critical steps in various metabolic processes [5], [6], [7], [8], [9], [10], [11], [25] and anti-PDI antibodies have been shown to also have pro and anti-thrombotic effects [26], [27]. However, the causal role of immunity in metabolic syndrome and particularly the role of anti-PDI antibodies needs to be further explored.

Our study suffers from several important limitations. First, our data suggest that post-streptococcal immune changes remain relatively stable across time, lasting years, but this will need to be further studied following documented streptococcal infections. Additionally, the prevalence of metabolic syndrome in our cohort is higher than usually reported and may reflect oversampling of habitual snorers, among whom there is a higher prevalence of metabolic syndrome. Other limitations of the current study are limited information on other confounding factors for metabolic syndrome such as diet and exercise habits.

Finally, since metabolic syndrome is a leading cause of morbidity and mortality in the developed world, and these study findings could be of importance for public health, independent replications of the results in other populations by other research groups are definitely needed.

Indeed, if replicated, our results may also have implications for the prevention of metabolic syndrome. The first-line interventions to reduce metabolic risk factors are lifestyle changes including weight loss, increased physical activity and healthy eating habits. While these are recommended for the entire population, identifying specific risk groups, such as those with post-streptococcal immunity, may lead to targeted approach and better compliance.
